# Metabolism and oxygen tension in Alzheimer’s disease mouse model using two-photon FLIM-PLIM microscopy

**DOI:** 10.1117/1.NPh.13.3.035008

**Published:** 2026-09-11

**Authors:** Vijay Kumar Sagar, Horst Wallrabe, Shagufta Rehman Alam, Evelyn Pardo, Wolfgang Becker, Andrés Norambuena, Ammasi Periasamy

**Affiliations:** a University of Virginia, The W.M. Keck Center for Cellular Imaging, Charlottesville, United States; b University of Virginia, Department of Biology, Charlottesville, United States; c Becker & Hickl, GmbH, Berlin, Germany; d University of Virginia, Department of Biomedical Engineering, Charlottesville, United States

**Keywords:** 2P-FLIM, 2P-PLIM, NAD(P)H, metabolism, ruthenium, oxygen tension, Alzheimer’s disease

## Abstract

**Significance:**

Alzheimer’s disease (AD) is a progressive neurodegenerative disorder characterized by cognitive decline and memory deficits, processes critically linked to mitochondrial dysfunction in the nervous system. Equally, adequate brain tissue oxygenation is essential for cerebral metabolism, and its impairment further compromises neuronal energy homeostasis. Together, mitochondrial dysfunction and disrupted cerebral oxygenation represent key interconnected pathological mechanisms underlying AD progression, highlighting the significance of targeting these pathways in understanding and managing the disease.

**Aim:**

Cerebral energy metabolism in the cortex of wild-type (WT) and Alzheimer’s disease (AD) model mice was investigated using minimally invasive two-photon FLIM, with the protein-bound fraction of nicotinamide adenine dinucleotide (phosphate) (NAD(P)H) a2% serving as a metric for mitochondrial metabolic activity. The probe “$\mathrm{Ru}(\mathrm{BPY}{)}_{3}$” was calibrated to examine the dynamic nature of its phosphorescence lifetime to estimate the partial pressure of oxygen (${\mathrm{pO}}_{2}$) in brain vasculature of live animals.

**Approach:**

Simultaneous two-photon fluorescence and phosphorescence lifetime imaging (2P-FLIM-PLIM) provides a minimally invasive approach to measure the metabolic state in combination with the ${\mathrm{pO}}_{2}$ in the vascularity of live animals. The metabolic state is obtained via FLIM of NAD(P)H, ${\mathrm{pO}}_{2}$ via the phosphorescence lifetime of $\mathrm{Ru}(\mathrm{BPY}{)}_{3}$, an oxygen-sensitive exogenous probe.

**Results:**

AD mouse cortices exhibit higher level of NAD(P)H a2% and lower level of ${\mathrm{pO}}_{2}$ compared with WT at anesthetized resting-state. 2P-PLIM investigation reveals that the lower level of ${\mathrm{pO}}_{2}$ in vascular network Alzheimer’s disease (AD) mice cortex indicates increased oxygen consumption in AD.

**Conclusions:**

The concurrent application of simultaneous 2P-FLIM-PLIM in the mouse brain establishes this dual-modality protocol for the simultaneous assessment of cerebral metabolic alterations using biomarker NAD(P)H and ruthenium-based cerebrovascular oxygen tension measurements. These findings underscore the utility of this multiparametric optical imaging strategy in elucidating the interplay between mitochondrial metabolic dysfunction and impaired brain tissue oxygenation, providing a compelling platform for advancing mechanistic investigations into the neuropathological sequelae of Alzheimer’s disease.

## 1 Introduction

The human brain represents only 2% of the body’s weight but uses ∼20% of total energy requirements.^1^ Neurons therefore have a critical requirement for a well-functioning energy supply to drive the central nervous system.^2^ Cellular energy is predominantly derived from adenosine triphosphate (ATP) produced via oxidative phosphorylation through the mitochondrial electron transport chain (ETC), with cytosolic glycolysis serving as a secondary, less efficient pathway of ATP generation.^3^^,^^4^ The progressive cognitive decline and memory deficit aspect of neurodegenerative disorders such as Alzheimer’s disease (AD) is *inter alia* linked with mitochondrial dysfunction in the nervous system.^5^ Biomolecules nicotinamide adenine dinucleotide phosphate (NAD(P)H) and flavin adenine dinucleotide (FAD) are involved in many metabolic reactions associated with glycolysis, oxidative phosphorylation (OXPHOS), and fermentation.^6^ Based on the auto-fluorescent nature of NAD(P)H, fluorescence lifetime imaging (FLIM) serves as a minimally noninvasive, label-free method to investigate metabolism at the cellular level, by measuring the fluorescence decay time of free/bound NAD(P)H coenzymes.^7^^–^^10^ This biomarker has been used to investigate mitochondrial function in animal models with respect to AD pathology.^6^^,^^9^^,^^11^^,^^12^ Brain tissue oxygenation is another important process for cerebral metabolism.^13^ During periods of adequate oxygenation, NAD(P)H is rapidly oxidized to $\mathrm{NAD}(P{)}^{+}$ as mitochondrial efficiently produces ATP through oxidative phosphorylation.^6^ Conversely, during hypoxic or anoxic conditions, the electron transport chain becomes impaired, leading to NAD(P)H accumulation as it cannot be effectively oxidized due to insufficient oxygen availability.^14^ Using multiparametric photoacoustic microscopy imaging through a cranial window, we quantified hemoglobin oxygen saturation (${\mathrm{sO}}_{2}$) simultaneously in cortical arteries and veins, with the arteriovenous ${\mathrm{sO}}_{2}$ difference serving as a measure of oxygen extraction fraction and thus tissue oxygen consumption.^11^

Multiple techniques have demonstrated their capabilities for measuring brain oxygenation, each with distinct trade-offs in spatial resolution and specificity. F18-fluorodeoxyglucose Positron Emission Tomography measures glucose uptake as a metabolic surrogate but does not directly measure oxygen,^15^ whereas O15-PET quantifies cerebral metabolic rate of oxygen directly but requires cyclotron-produced isotopes and offers poor spatial resolution.^16^ Noninvasive approaches such as near-infrared spectroscopy estimate hemoglobin oxygenation but with limited spatial resolution,^17^ whereas electron paramagnetic resonance oximetry measures tissue ${\mathrm{pO}}_{2}$ directly but requires implantation of paramagnetic probes.^18^ Blood oxygen level-dependent functional MRI detects deoxyhemoglobin changes but reflects a complex convolution of blood flow, volume, and oxygenation.^19^ Multiparametric photoacoustic microscopy, by contrast, provides a label-free, high-resolution approach to simultaneously quantify ${\mathrm{sO}}_{2}$ and blood flow in cortical vessels,^11^ whereas PLIM complements this by directly measuring ${\mathrm{pO}}_{2}$ at the cellular and subcellular level through collisional quenching of oxygen-sensitive phosphorescent probes.^20^^–^^26^ The quantitative understanding of metabolism and oxygen tension in biological specimens demands minimally noninvasive and reliable imaging platforms.

Here, we report on the application of a dual 2P FLIM-PLIM system to investigate mitochondrial metabolic activity (by FLIM of NAD(P)H) simultaneously with oxygen consumption $(\mathrm{Ru}(\mathrm{BPY}{)}_{3}$) and ${\mathrm{pO}}_{2}$ in the live mouse brain.

## 2 Materials and Methods

### 2.1 Materials

Tris(2,2′-bipyridyl)dichlororuthenium(II) hexahydrate ($\mathrm{Ru}(\mathrm{BPY}{)}_{3}$) was purchased from Sigma-Aldrich, Saint Louis, USA. The nutrient amino acid (stimulating metabolism) mixture was prepared by mixing arginine (R) and leucine (L) at 100  μM final concentration in Hank’s balanced salt solution (HBSS). Isoflurane was purchased from MWI Veterinary Supply Co.

### 2.2 Animal Models

The PS19 tauopathy and wild type mouse models (3 months old) were purchased from The Jackson Laboratory.^27^ Three-month-old PS19 mice were selected as this age represents a presymptomatic stage with measurable mitochondrial metabolic alterations prior to overt synaptic and cognitive deficits.^28^ All surgical and animal handling procedures were performed as approved under the guidelines for Animal Care & Use Committee (ACUC) and the guidelines of the University of Virginia (UVA).

### 2.3 Cranial Window and Tail Vein Injection Preparation

**Fig. 1:**
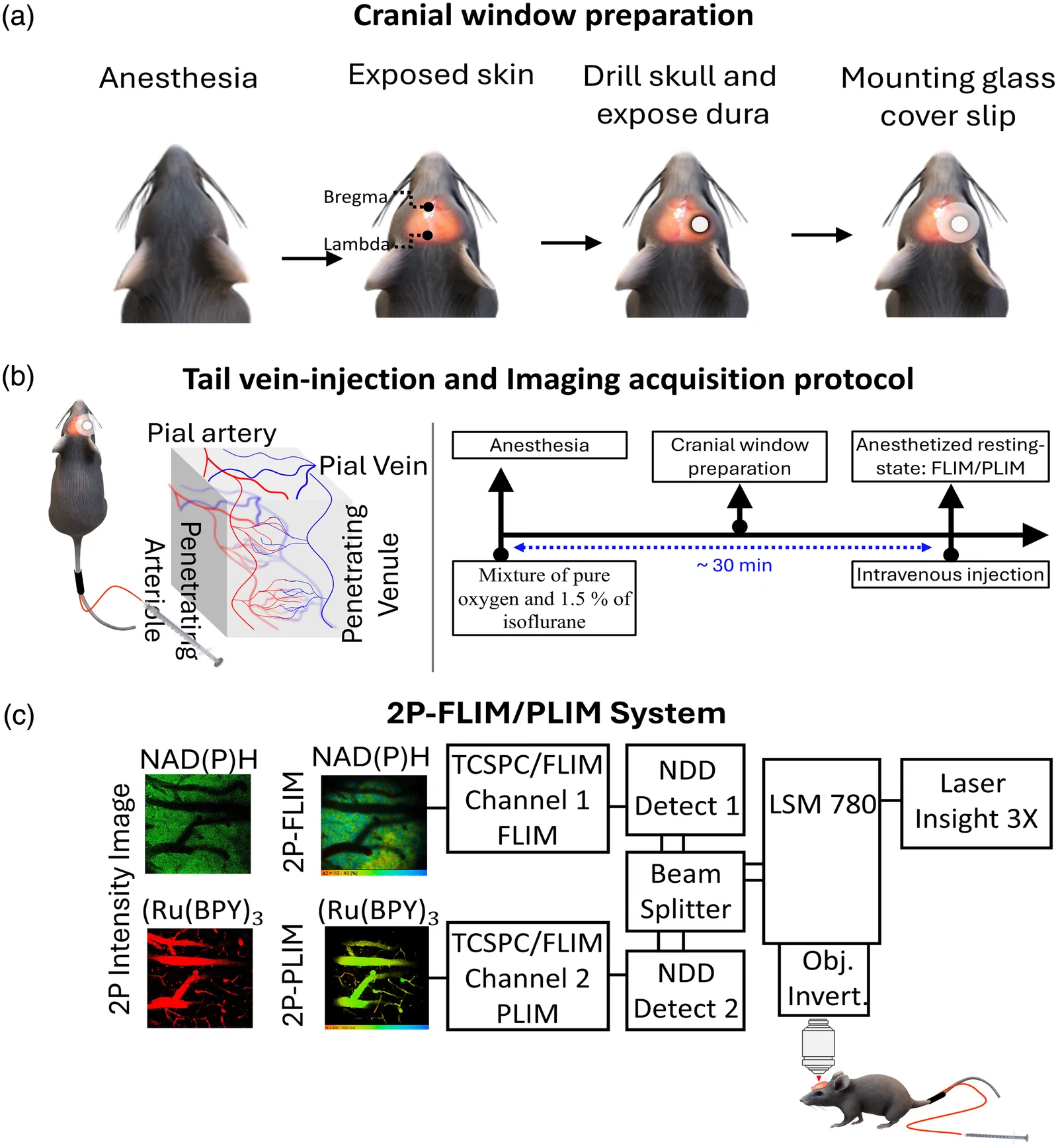
Schematic illustration for *in vivo* animal preparation before imaging and 2P-FLIM-PLIM instrumentation. (a) The cranial window preparation; (b) cortical vascular anatomy and tail-vein injection/imaging acquisition protocol. (Left) Schematic of cortical vascular anatomy showing pial arteries, pial veins, and penetrating arterioles/venules, used for vessel-type identification throughout this study. (Right) Imaging acquisition protocol: mice were anesthetized with 1.5% isoflurane in pure oxygen, followed by cranial window preparation and a∼30  min recovery period prior to intravenous probe injection and 2P-FLIM-PLIM acquisition under an anesthetized, resting state. (c) Schematic of simultaneous 2P-FLIM-PLIM image acquisition showing TCSPC channel 1 (FLIM) and channel 2 (PLIM) detected by nondescanned detectors (NDD). Representative 2P intensity images of NAD(P)H autofluorescence (before $\mathrm{Ru}(\mathrm{BPY}{)}_{3}$ injection) and ruthenium phosphorescence (after $\mathrm{Ru}(\mathrm{BPY}{)}_{3}$ injection) were acquired using the standard two-photon detection channel, whereas the corresponding color-coded FLIM and PLIM lifetime images were recorded via the NDD channels.

Figure 1 shows a schematic illustration of the cranial window preparation (a), tail vein injection (b), and distribution of ${\mathrm{pO}}_{2}$ (c). Live mice were anesthetized in an induction chamber with a mixture of pure oxygen and 1.5% of isoflurane. A temperature-controlled heating pad (37°C) was used to prevent hypothermia after anesthesia and before surgery. Animals under anesthesia were kept on a heating pad, and a rodent mask was applied to deliver a continuous flow of a mixture of oxygen and isoflurane. Sterilized surgical devices were used throughout, and cranial hair was removed by depilatory cream. The exposed skin was removed, and skull was thinned with a miniature drill without damaging the dura. The skull thinning procedure represents a minimally invasive optical access approach, preserving dural integrity while providing sufficient optical transparency for two-photon excitation microscopy of the cortical vasculature. Phosphate-buffered saline (PBS) was applied on the cranial window, covered with a glass cover slip to seal the microenvironment. To image cerebral oxygen levels in blood vessels with 2P excitation, 100  μL (0.5 mM) phosphorescent $(\mathrm{Ru}(\mathrm{BPY}{)}_{3}$ dye was applied by tail vein injection, as shown in Fig. 1(b).

### 2.4 2P-FLIM and PLIM Imaging

For live imaging sessions, anesthesia was maintained via continuous delivery of isoflurane (1.5% to 2% v/v) in oxygen through a fitted rodent nose cone, and core body temperature was sustained at 37°C using a homeothermic heating pad. The complete surgical preparation, including anesthesia induction, cranial window preparation, and transfer to the imaging stage, took ∼30  min prior to image acquisition, allowing sufficient time for stabilization of isoflurane-induced hemodynamic effects. Respiratory rate and depth were monitored visually throughout each imaging session to confirm physiological stability. PBS (pH 7.4) was applied to the cranial window surface and sealed with a glass coverslip to maintain tissue hydration, minimize motion artifacts, and ensure a stable optical interface for 2P imaging. Images were acquired at a cortical depth of 120 to 150  μm below the cortical surface. The aim for simultaneous FLIM-PLIM was to acquire images of the metabolic state and the ${\mathrm{pO}}_{2}$ at the parietal mouse cortex, showing cortical tissue with major and minor vasculature. Seeking vasculature served two aims, (i) as fiduciary markers to ensure identical Fields-of-View (FoV) and (ii) for tracking PLIM of $\mathrm{Ru}(\mathrm{BPY}{)}_{3}$. Figure 1(c) provides a schematic of the 2P FLIM-PLIM instrument. For *in vivo* metabolic as well as oxygen tension measurement, an objective inverter (InverterScope^®^, LSM Tech, Inc;^29^) was used to convert the inverted confocal microscope (Zeiss LSM 780) for upright applications. 2P excitation was obtained by a Chameleon Vision-II (Coherent Inc.) ultrafast Ti:sapphire laser (690 to 1060 nm, 80 MHz, 150 fs). For metabolic imaging, an ET450/50 emission band-pass filter (Chroma Technologies Corp.) was used, and for the $\mathrm{Ru}(\mathrm{BPY}{)}_{3}$, channel a long pass filter (LP560) was used. Both signals were captured via the nondescanned detection path of the microscope system and detected by HPM-100-40 single-photon hybrid detectors (Becker & Hickl, GmbH).^30^

The detector pulses are processed by two channels of a three-channel SPC-153 TCSPC/FLIM system (Becker & Hickl, GmbH).^30^^,^^31^ Images were acquired using a Zeiss Plan-Apochromat 10×/0.45 objective (Plan-Apochromat) with 2P excitation at 800 nm, providing a theoretical lateral resolution of ∼1.2  μm and axial resolution of ∼9  μm (lateral: 0.7 λ/NA; axial: 2.3 λ/NA^2^), sufficient to resolve individual cortical vascular compartments including capillaries (3 to 5  μm diameter). To obtain sufficient photons per pixel at the cortical focal plane, the average excitation laser power at the specimen plane was maintained ∼120  mW with the collection time of 180 s, without evidence of cortical tissue photobleaching. Excitation laser power was carefully optimized prior to experimental imaging. We showed power-dependent assessment of cortical tissue integrity which revealed visible photobleaching at ∼140  mW at the objective output (see Fig. S1 in the Supplementary Material). The effective power at the focal plane is further attenuated relative to the objective output power due to absorption and scattering by the overlying cortical tissue, providing an additional safety margin against phototoxicity.

We have chosen $\mathrm{Ru}(\mathrm{BPY}{)}_{3}$ due to its photochemical and chemical stability, low cytotoxicity along with 2P excitation capability for deeper tissue penetration and higher spatial resolution.^32^^,^^33^ Due to the excitation mechanism of PLIM (intersystem crossing and emission from the triplet state) a single femtosecond laser pulse does not generate enough excited-state population.^21^ The system therefore uses on/off modulation of the laser pulse train via the acousto-optic modulator (AOM) of the microscope. During the on phase of the laser FLIM is recorded and phosphorescence is built up, during the OFF-phase the phosphorescence decay of $\mathrm{Ru}(\mathrm{BPY}{)}_{3}$ (lifetime 0.3 to 0.8  μs is recorded).^24^^,^^34^ The modulation signal was generated by a B&H DDH-210 pulse generator module. The module is controlled by general SPCM FLIM-PLIM software. To synchronize the ON/OFF signal the DDG-210 was triggered by the pixel clock signal of the LSM 780 scan controller. The output of the DDG-210 was connected to the PLIM” input of the LSM 780 microscope. This input is optional for the LSM 780 and has to be enabled by a “PLIM MACRO”. The pixel dwell time of 12  μs and laser repetition rate of 80 MHz, ∼960 excitation cycles were acquired per pixel per frame pass. The laser was gated ON for 2  μs for FLIM excitation and OFF for the remaining 10  μs for simultaneous PLIM phosphorescence collection.

## 3 Data Processing and Analysis

**Fig. 2:**
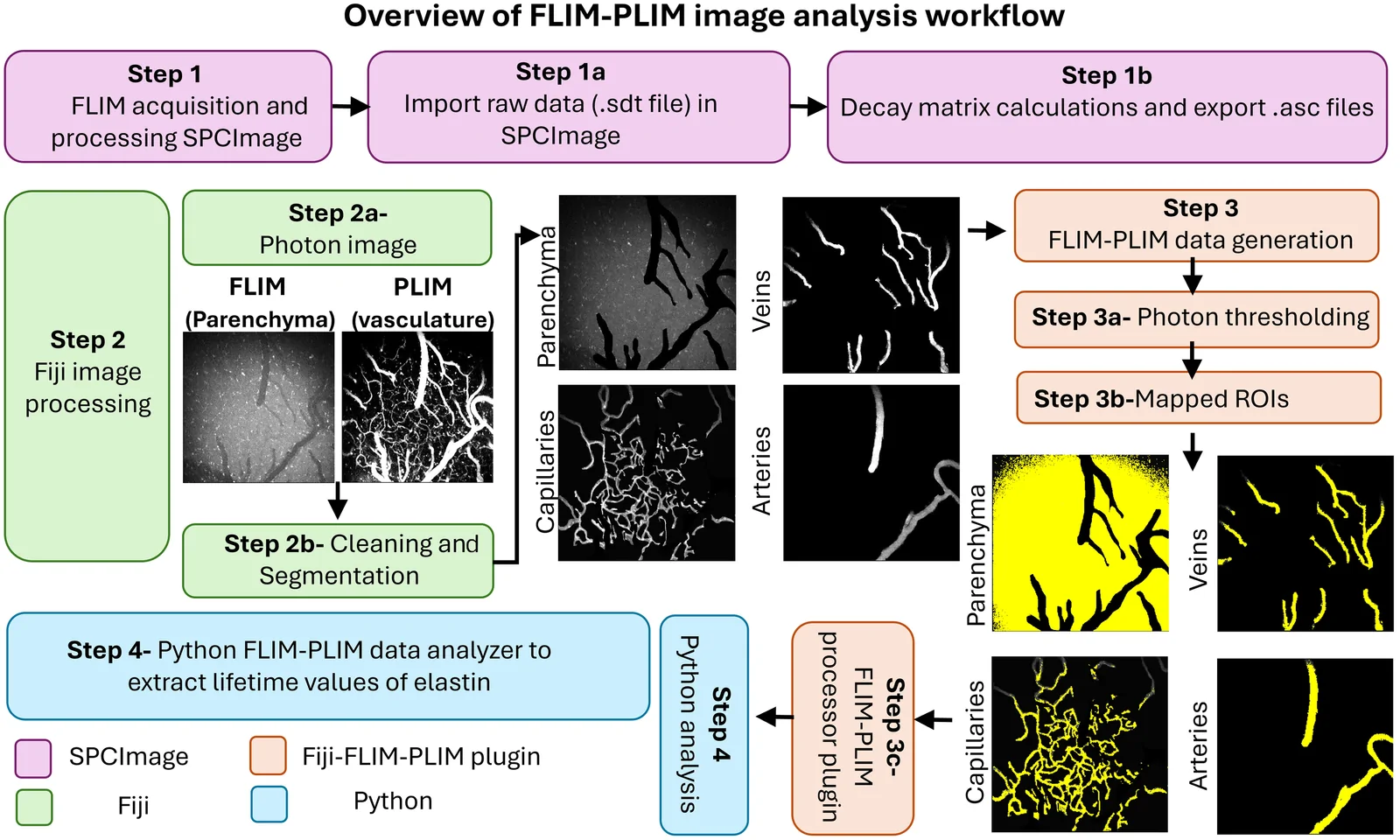
Overview of the FLIM-PLIM image analysis workflow. The FLIM-PLIM data processing pipeline requires *.sdt files (steps 1a and 1b) to compute decay matrices. Photon images were preprocessed (step 2b) by manual cleaning and morphology-based segmentation to isolate signals from the parenchyma, capillaries, veins, and arteries. Photon thresholding (step 3a) was applied to the segmented photon images to select pixel-level data using a 1×1 ROI selection. The resulting mapped ROIs are shown in step 3b. A custom-built FLIM-PLIM processor plugin (step 3c) was used to extract and generate the FLIM-PLIM lifetime data from the selected ROIs. Custom Python scripts (step 4) were used to represent the a2% NAD(P)H distribution from mitochondria and the ruthenium lifetime ($\tau_{1}$) distribution across the selected ROIs.

Overview of FLIM/PLIM image analysis workflow is given in Fig. 2. FLIM and PLIM data were processed separately using SPCImage software (v 9.0 NG), Fiji and Python.

### 3.1 FLIM Data Analysis (NAD(P)H)

2P-FLIM data analysis is described in reference.^8^ In brief, 2P-FLIM data (B&H data file “.sdt file”) were imported into SPCImage software (v 9.0 NG) (step 1). To run the decay analysis, we used a two-component decay model in combination with a synthetic IRF. The two-component decay model is defined as $I(t)=a_{1}.\exp(-\frac{t}{\tau_{1}})+a_{2}.\exp(-\frac{t}{\tau_{2}})$, where the bound fraction is $a_{2}\%=a_{2}/(a_{1}+a_{2})\times 100$. The fit was performed using maximum-likelihood estimation (MLE). MLE delivers near-ideal fit accuracy down to extremely low photon counts. Nevertheless, the results are often noisy due to low fluorescent signals from the autofluorescence of the specimens. To improve the number of photons per pixel, the ’binning’ function of SPCImage NG was used. The function combines the decay data of adjacent pixels without combining the intensity data. It thus improves the signal to noise ratio (SNR) of the decay data without compromising spatial resolution. The binning function of SPCImage NG is controlled by the “Bin” parameter Bin=2 was selected to obtain sufficient photons. The fit-accuracy indicator, $\chi^{2}$, remained close to 1, indicating accurate fitting. A decay matrix was calculated and generated for NAD(P)H by the software (step 2). Color coded 2P FLIM fitted images of enzyme bound fraction of NAD(P)H for WT and PS19 were generated using SPCImage software (v9.0 NG). We focus on $a_{2}\%$ as our primary metabolic readout because $\tau_{1}$ and $\tau_{2}$ remain relatively stable across metabolic conditions, whereas $a_{2}\%$ varies sensitively with the shift between glycolytic and oxidative metabolism. A higher $a_{2}\%$ indicates a greater proportion of enzyme-bound NAD(P)H, reflecting more oxidative mitochondrial activity.

### 3.2 PLIM Data Analysis of Ru(BPY)_3_

For 2P-PLIM data analysis, acquired data (“.sdt file”) was imported into SPCImage NG (v 9.0 NG) (step 1). Characterization of $\mathrm{Ru}(\mathrm{BPY}{)}_{3}$ in solution was lifetime-fitted by single-component analysis, incomplete multi-exponential decay model and maximum likelihood estimation (MLE) at bin 3 for sufficient photons. The ‘laser repetition’ parameter in case of PLIM data is the period of the ON/Off modulation. It was set to 12  μs, corresponding to the pixel rate. For mouse image analysis, bi-exponential/two-component fitting was used and the incomplete multi-exponential decay model, MLE, laser repetition, bin 3 was maintained. The parameter “offset” was allowed to float and “scatter” was at default zero. The fit-quality parameter ‘$\chi^{2}$’ with these settings remained close to 1. In contrast to FLIM data, the IRF in the PLIM data is the waveform of laser modulation rather than convolution of laser pulse with the detector response. The rectangle function or alternative delta function can be used as an IRF to fit the PLIM data; there was no noticeable difference in the final outcomes. After optimizing the fitting parameters, the decay matrix corresponding to $\mathrm{Ru}(\mathrm{BPY}{)}_{3}$ lifetime was calculated (step 2).

### 3.3 Fiji and Python Processing

Photon (intensity) images (step 2) exported from SPCImage were imported into Fiji (ImageJ, NIH) for manual segmentation of cortical regions of interest (ROIs). Unwanted regions (step 2b), including out-of-focus areas, tissue boundaries, and nontarget structures, were manually delineated and excluded from analysis by assigning a pixel intensity of zero. Vascular segmentation into arteries, veins, and capillaries was guided by established morphological criteria. Arteries were identified by elastin autofluorescence under 2P excitation, whereas veins were distinguished by vessel diameter, low intrinsic contrast, and branching morphology. Capillaries, not readily discernible in label-free photon images, were delineated following intravenous ruthenium administration, whose phosphorescence signal in the simultaneously acquired PLIM images provided sufficient intravascular contrast to resolve the capillary network. Following manual cleaning, the pre-processed images were analyzed by a custom Java plugin in Fiji (step 3a), producing results by pixel regions-of-interest (ROIs) within each field-of-view (FoV). ROI selection by 1×1 ROI pixel size is based on intensity threshold to capture the more prominent, metabolically active NAD(P)H auto-fluorescent mitochondrial signals for FLIM data and $\mathrm{Ru}(\mathrm{BPY}{)}_{3}$ signal for vasculature areas for PLIM data. Following intensity thresholding, mapped ROIs (step 3b) were generated by projecting pixels meeting the defined photon count criteria onto the segmented cortical regions, producing binary masks that spatially delineated the retained pixels within each vascular compartment (arteries, veins, and capillaries) and parenchyma. These mapped ROIs represent the final set of pixels selected for fluorescence lifetime analysis, with excluded regions rendered as zero-intensity background. The resulting ROI masks were subsequently processed using a custom built FLIM-PLIM processor Fiji plugin (step 3c), which extracted per-pixel lifetime parameters, including $\tau_{1}$, $\tau_{2}$, a1%, and a2% from each segmented compartment independently. Each vascular compartment and cortical ROI was processed separately through the plugin, ensuring that lifetime parameter distributions were extracted exclusively from the spatially defined regions of interest, free from signal contributions of nontarget structures. Following PLIM data processing, ruthenium lifetime values were filtered *post hoc* to retain only physiologically plausible estimates within the expected lifetime range for each vascular compartment. These lifetime windows were calculated directly from the *in vitro* Stern-Volmer calibration constants $\tau_{0}$ and $k_{q}$ using the rearranged equation $\tau_{1}=\tau_{0}/(1+k_{q}\cdot {\mathrm{pO}}_{2})$. Specifically, lifetime values were retained within the following $\tau_{1}$ windows: arteries 313 to 357 ns (corresponding to expected ${\mathrm{pO}}_{2}$ of 80 to 100 mmHg), capillaries 357 to 500 ns (expected ${\mathrm{pO}}_{2}$ of 20 to 60 mmHg), and veins 417 to 526 ns (expected ${\mathrm{pO}}_{2}$ of 15 to 40 mmHg). Lifetime values outside these ranges were excluded prior to ${\mathrm{pO}}_{2}$ calculation. The same filtering criteria were applied consistently across all WT and PS19 animals.

The extracted per-pixel lifetime parameters were subsequently imported into Python for statistical analysis and data visualization. Python custom codes (step 4) and Microsoft Excel were used to generate data plots. Error bars represent +/− s.e.m. (n=5 for WT and n=4 for PS19).

## 4 Phosphorescence Lifetime Characterization and Calibration of Ru(BPY)_3_

### 4.1 Experimental Set-up for Calibration

**Fig. 3:**
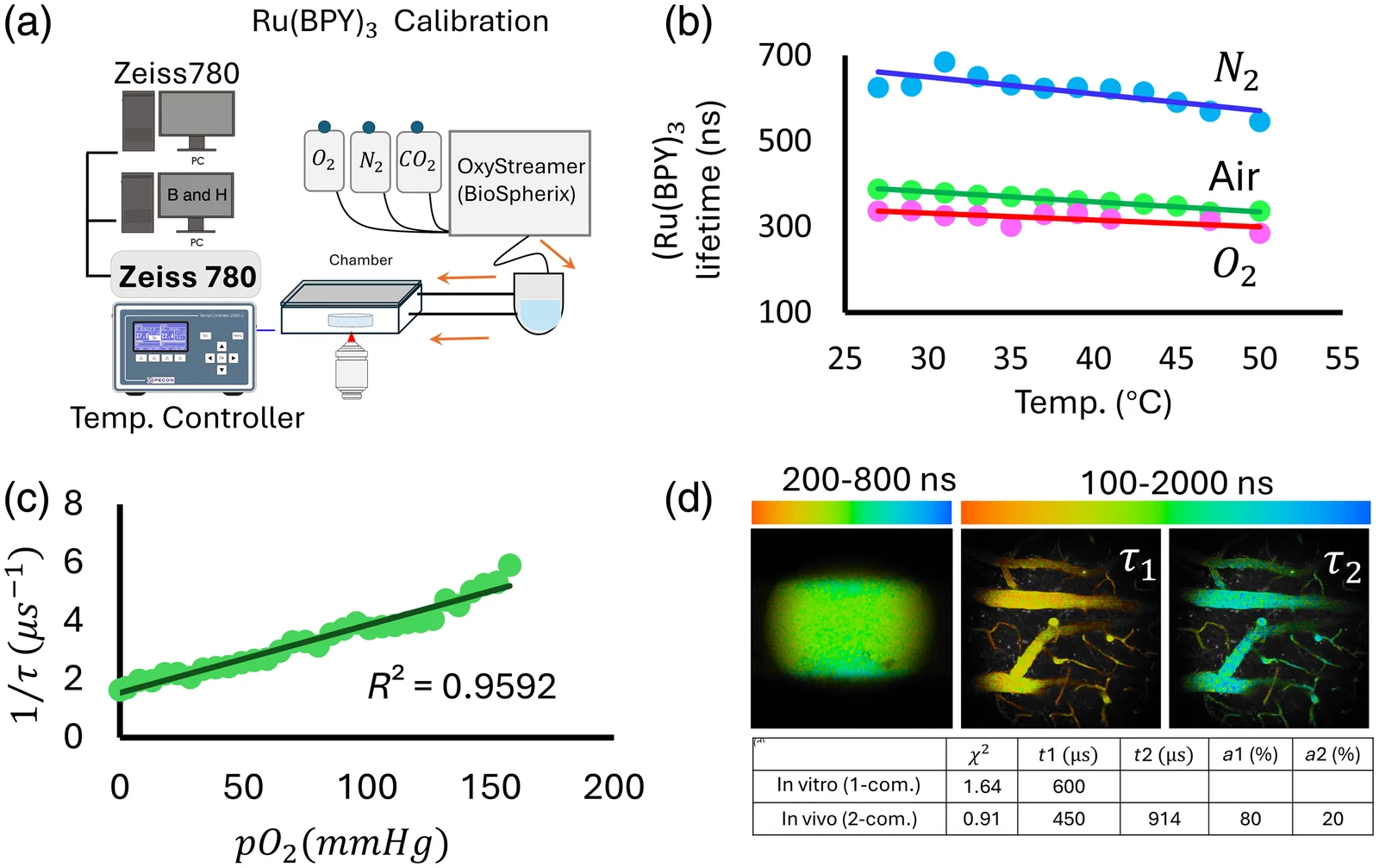
$\mathrm{Ru}(\mathrm{BPY}{)}_{3}$ lifetime calibration. The experimental setup for the calibration of $\mathrm{Ru}(\mathrm{BPY}{)}_{3}$ is shown in (a). $\mathrm{Ru}(\mathrm{BPY}{)}_{3}$’s lifetime as a function of temperature under $N_{2}$, air, and $O_{2}$ (b). The inverse of $\mathrm{Ru}(\mathrm{BPY}{)}_{3}$’s lifetime as a function of partial pressure of oxygen (${\mathrm{pO}}_{2}$) at 37°C (c). The color-coded PLIM images of $\mathrm{Ru}(\mathrm{BPY}{)}_{3}$ solution in capillary and $\tau_{1}$ and $\tau_{2}$ components PLIM images of mouse cortex along with the *in vitro* and *in vivo* fitted PLIM parameters are shown in panel (d).

The experimental set-up for the 2P lifetime characterization and calibration of $\mathrm{Ru}(\mathrm{BPY}{)}_{3}$ is provided in Fig. 3(a). The calibration method was adopted from the published report with slight modifications.^34^ The OxyStreamer (BioSpherix^35^) controls the flow of $N_{2}$, ${\mathrm{CO}}_{2}$, and $O_{2}$ gases. Temperature-control enclosed chamber (Pecan TempController 2000 to 2), inserted in 2P FLIM-PLIM Zeiss LSM780 microscope system stage was used to control the temperature. $\mathrm{Ru}(\mathrm{BPY}{)}_{3}$ at 100  μM concentration was used to calibrate the phosphorescence lifetime.

### 4.2 Dynamic Behavior of Ru(BPY)_3_ Lifetime in the Local Microenvironment of Live Animal Model

$\mathrm{Ru}(\mathrm{BPY}{)}_{3}$’s lifetime is sensitive to the local microenvironment which can influence the interpretation in *in vivo* imaging. To investigate the variability in lifetime in temperature-dependent and controlled gaseous (oxygen, nitrogen, air), additional studies were performed. The phosphorescence lifetime ($\tau =1/(k_{r}+k_{nr})$) is inversely proportional to all the decay rates. As the temperature increases, nonradiative decay rate becomes dominant which leads to shortening of the lifetime. Moreover, in gaseous environments, dynamic (collisional) quenching by external molecules also increases as the temperature rises which consequently reduces the lifetime. Oxygen-dependent lifetime quenching of $\mathrm{Ru}(\mathrm{BPY}{)}_{3}$ helps to estimate the Stern-Volmer quenching constant, important as a crucial parameter for $\mathrm{Ru}(\mathrm{BPY}{)}_{3}$ as an optical sensor to investigate oxygen tension in live animals. Moreover, the total positive charge on $\mathrm{Ru}(\mathrm{BPY}{)}_{3}$ facilitates its attraction to negatively charged surfaces (blood vessels) as well as binding to negatively charged biomolecules (DNA) via weak electrostatic interactions.^36^

To investigate the role of local microenvironment on $\mathrm{Ru}(\mathrm{BPY}{)}_{3}$ lifetime, three separate measurements were performed in nitrogen (i), oxygen (ii), and air (iii) environments as a function of temperature. For the temperature dependence, an enclosed chamber was maintained at 27°C and then heated at 2°C/min intervals to collect data. Figure 3(b) shows the temperature-dependent lifetime of $\mathrm{Ru}(\mathrm{BPY}{)}_{3}$ in three different local environments.

(i) For local inert environment ($N_{2}$), in an enclosed chamber, $\mathrm{Ru}(\mathrm{BPY}{)}_{3}$ solution was sparged with compressed $N_{2}$ gas (bubbled directly into the sample) for 40 min at 37°C before starting the measurement. This period is necessary to maintain the equilibrium concentration of oxygen in presence of $N_{2}$ during the experiment.^34^ Once the equilibrium was reached, temperature-dependent PLIM data were recorded at heating rate of 2°C/min in presence of $N_{2}$.
(ii) For oxygen-dependent measurements, $N_{2}$ gas was maintained in the chamber and $O_{2}$ gas at 13 mmHg was bubbled directly into the sample for 40 min. Once the equilibrium was reached, temperature dependent PLIM data were recorded at heating rate of 2°C/min.
(iii) At the atmospheric conditions (air), the experiments were performed without stirring any gas in the solution as a function of temperature.
(iv) Electrostatic interactions of $\mathrm{Ru}(\mathrm{BPY}{)}_{3}$ in the blood stream are highly dependent on its total electrostatic charge. The net positive charge can lead to electrostatic interactions with biomolecules and other negatively charged surfaces on the blood vessels. The innermost layers of brain blood vessels (capillaries, arteries, and veins) are composed of endothelial glycocalyx, which are highly negatively charged. The interaction of positively charged $\mathrm{Ru}(\mathrm{BPY}{)}_{3}$ within the blood can influence its photophysical properties with the possibility of binding and uptake by the endothelial cells. $\mathrm{Ru}(\mathrm{BPY}{)}_{3}$ can bind to most abundant protein in blood plasma (serum albumin) with moderate-to-strong affinity as well as bipyridine ligands on $\mathrm{Ru}(\mathrm{BPY}{)}_{3}$ allows it to bind weakly and electrostatically with negatively charged phosphate backbone of DNA.^37^^,^^38^ It has also been reported that $\mathrm{Ru}(\mathrm{BPY}{)}_{3}$ does not exhibit strong affinity for red blood cells. Apart from the electrostatic net positive charge, small molecular size allows ruthenium complex to pass through gaps or pores between endothelial cells in the blood vessels.

### 4.3 Estimation of Bio-Molecular Quenching Constant

Quantitative fluorescence lifetime quenching in presence of oxygen is an analytical approach to measure the concentration of molecular oxygen. The quenching behavior is expressed by Stern-Volmer (S-V) equation,

$$\frac{\tau_{0}}{\tau }=1+K_{\mathrm{SV}}.[O_{2}],$$ (1)

where $\tau_{0}$ is the phosphorescence lifetime in the absence of oxygen, τ is the phosphorescence lifetime in presence of oxygen, and oxygen concentration is given by $\left[O_{2}\right]$. $K_{\mathrm{SV}}$ is the Stern-Volmer quenching constant which is a product of phosphorescence lifetime in the absence of oxygen and the bimolecular quenching rate constant $\left(K_{SV}=k.\tau_{0}\right)$. The oxygen content was converted into partial pressure of oxygen units (mmHg) using Henry’s law which is defined as the amount of gas (oxygen) dissolves in a liquid is directly proportional to the partial pressure of that gas (oxygen) above the liquid, where α is oxygen solubility coefficient

$$[O_{2}]=\;\alpha \times \;{\mathrm{pO}}_{2}.$$ (2)

Equation (1) can be rearranged as

$$\frac{\tau_{0}}{\tau }=1+k.\tau_{0}\times \alpha \times {\mathrm{pO}}_{2}.$$ (3)

To estimate the bio-molecular quenching constant, a separate experiment was performed [Fig. 3(c)]. In an enclosed chamber, a well -stirred solution of $\mathrm{Ru}(\mathrm{BPY}{)}_{3}$ (100  μM) was sparged with compressed $N_{2}$ gas into the solution for 40 min. The experiment was performed at the constant temperature of 37°C. In complex biological systems $\mathrm{Ru}(\mathrm{BPY}{)}_{3}$ exhibits bi-exponential decay. PLIM data in the absence of oxygen was recorded to estimate the maximum lifetime of $\mathrm{Ru}(\mathrm{BPY}{)}_{3}$ in absence of oxygen. Another tube from the OxyStreamer was connected to the chamber to deliver a low concentration of $O_{2}$ (∼2.58  mmHg). PLIM data sets at various concentrations of oxygen were recorded. A linear decline in the phosphorescence lifetime of PLIM data was observed between 0 and 158 mmHg in the presence of $O_{2}$. The relationship between phosphorescence lifetime and oxygen partial pressure [Fig. 3(c)] is described by the modified Stern-Volmer relation.^39^

$$\frac{\tau_{0}}{\tau }=1+k_{q}.\tau_{0}.{\mathrm{pO}}_{2},$$ (4)

where $k_{q}(=k\alpha )$ is expressed in units of ${\mathrm{mmHg}}^{-1}\;s^{-1}$. The estimated constant ($k_{q}$) was $2\times 10^{-6}\;\mathrm{mm}{\mathrm{Hg}}^{-1}\;{\mathrm{ns}}^{-1}$ and $\tau_{0}=667\;\mathrm{ns}$ ($R^{2}=0.96$). The partial pressure of oxygen (${\mathrm{pO}}_{2}$) was estimated using the above relation as follows:

$${\mathrm{pO}}_{2}=\frac{1}{k_{q}}(\frac{1}{\tau }-\frac{1}{\tau_{0}}).$$ (5)

The 2-components of $\mathrm{Ru}(\mathrm{BPY}{)}_{3}$ fluorescence lifetime indicates free and bound nature of ruthenium complex to the biomolecules. The acquired 2P-PLIM images were fitted by the SPCImage software v9.0 NG (Becker & Hickl GmbH). The fitted color-coded PLIM images are in Fig. 3(d) and indicate the distinct lifetimes of $\mathrm{Ru}(\mathrm{BPY}{)}_{3}$ in solution as compared with *in vivo*. Two-component PLIM parameters in mouse cortex can be assigned to quenched lifetime $\left(\tau_{1}\right)$ of $\mathrm{Ru}(\mathrm{BPY}{)}_{3}$ molecule in presence of oxygen molecules and unquenched lifetime $\left(\tau_{2}\right)$ of $\mathrm{Ru}(\mathrm{BPY}{)}_{3}$ molecule.

## 5 Results

### 5.1 Metabolic Status and Oxygen Tension in WT and PS19 Mouse Cortices

Here, $\mathrm{Ru}(\mathrm{BPY}{)}_{3}$ was applied to track oxygen levels in combination with NAD(P)H FLIM protocols.^40^ Here, $\mathrm{Ru}(\mathrm{BPY}{)}_{3}$ is applied to track oxygen levels in combination with our FLIM NAD(P)H protocols. $\mathrm{Ru}(\mathrm{BPY}{)}_{3}$ experimental PLIM data plays an important role in providing oxygen-related metrics in WT and PS19 mouse models. This group has extensively published data on the effects of nutrient-induced metabolic pathways in neuronal cell models and live WT and cloned mouse models based on FLIM and biochemical [*in vitro*] experiments.^9^^,^^11^

**Fig. 4:**
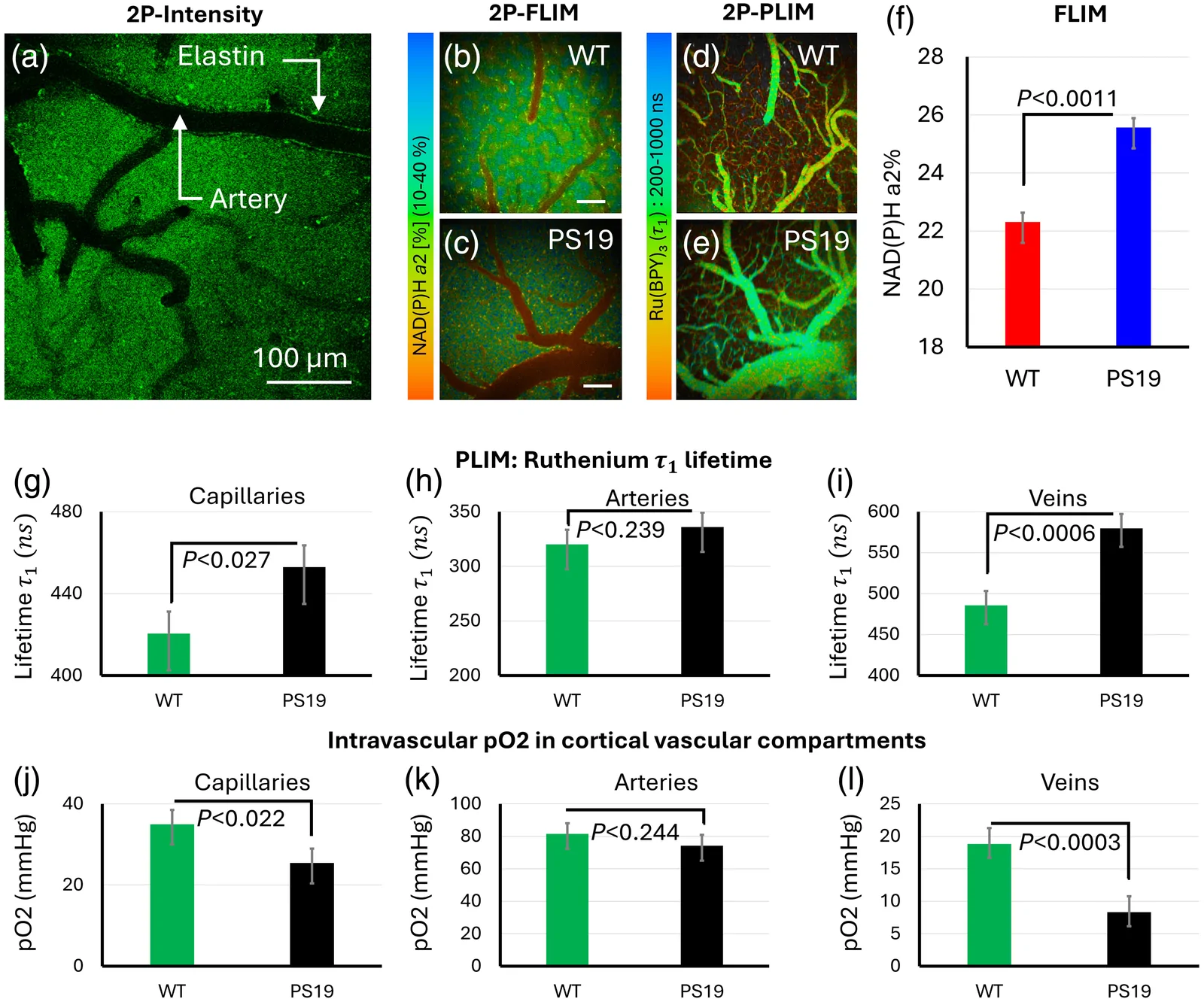
Simultaneous 2P-FLIM-PLIM imaging of WT and PS19 mouse cortices. 2P-intensity image of mouse cortex indicating elastin autofluorescence signature from artery in given in panel (a). Color coded FLIM images of enzyme-bound fraction of NAD(P)H (a2%) for WT and PS19 are given in panels (b) and (c). Color-coded PLIM images of $\mathrm{Ru}(\mathrm{BPY}{)}_{3}$ lifetime for WT and PS19 are given in panels (d) and (e). 2P-FLIM-PLIM parameter comparison of WT and PS19 mouse cortices in an anesthetized resting-state. (f) NAD(P)H a2% at baseline in WT and PS19 mice. (g)–(i) Ruthenium $\tau_{1}$ lifetime in capillaries (g), arteries (h), and veins (i). (j)–(l) Intravascular ${\mathrm{pO}}_{2}$, calculated from $\tau_{1}$ via the Stern-Volmer relationship, in capillaries (j), arteries (k), and veins (l). All comparisons (f)–(l) were performed using animal-level mean values (n=5 WT, n=4 PS19 mice) to account for the nested structure of pixel-level measurements, using Welch’s two-sample t-test. NAD(P)H a2% and $\tau_{1}$ were significantly elevated in PS19 relative to WT in capillaries and veins, and in NAD(P)H a2% overall, whereas arterial $\tau_{1}$ and ${\mathrm{pO}}_{2}$ did not reach statistical significance at this sample size. Correspondingly, ${\mathrm{pO}}_{2}$ was significantly reduced in PS19 capillaries and veins. Error bars represent ±SD across animals.

Arteries were identified by their bright elastin autofluorescence signal under 2P excitation^41^ [Fig. 4(a)], which was further confirmed by their lower $\mathrm{Ru}(\mathrm{BPY}{)}_{3}$ lifetime values in PLIM images, consistent with higher arterial oxygen content relative to veins (Figs. 4(d) and 4(e)]. Capillaries were delineated using $\mathrm{Ru}(\mathrm{BPY}{)}_{3}$ phosphorescence contrast as described in the Methods section. The conclusions on the differences between WT and PS19 are unchanged and based on identical conditions. The mouse parenchyma NAD(P)H autofluorescence (green) image [Fig. 4(a)] shows the bright elastin lining around the artery. NAD(P)H exists in free and bound states with characteristic ‘free’ lifetimes of 400 to 500 ps (short decay ($\tau_{1}$)) and 2 to 3 ns (a longer decay ($\tau_{2}$)) for “enzyme-bound” lifetime. Color-coded FLIM images of enzyme bound fractions NAD(P)H a2% of WT and PS19 mouse cortices are shown in Figs. 4(b) and 4(c). Two-components fitted color-coded 2P-PLIM images of $\tau_{1}$ for WT and PS19 mouse cortex are in Figs. 4(d) and 4(e).

### 5.2 Quantifying NAD(P)H a2% and ${\mathrm{pO}}_{2}$ Levels by Ruthenium Lifetime

The results compare the WT versus PS19 metabolic states of mouse cortical parenchyma under anesthetized resting-state conditions, assessed by NAD(P)H a2%. Vascular oxygen-related measurements are explored including ${\mathrm{pO}}_{2}$ under anesthetized resting-state conditions using PLIM microscopy. Under these conditions, PS19 showed a higher enzyme-bound fraction of NAD(P)H a2% in mouse cortical parenchyma compared with WT [Fig. 4(f)], indicating a higher overall mitochondrial metabolic activity. This altered brain metabolism compared with WT has been reported in the literature.^42^

Increases in the ruthenium probe lifetime signify a reduction in blood oxygen. The process of quantifying ${\mathrm{pO}}_{2}$ by ruthenium lifetime values is described in M&M. As described in the Methods, $\tau_{1}$ was used as the oxygen-sensitive parameter for ${\mathrm{pO}}_{2}$ quantification. Figures 4(g)–4(i) describes the changes in ruthenium $\tau_{1}$ lifetime of the total vasculature of the images.

Two-population means were compared using Welch’s two-sample t test (assuming unequal variances). Five WT and four PS19 mice were used, to exclude signal contributions from blood vessels and lipofuscin, thereby isolating mitochondrial NAD(P)H fluorescence from cortical neurons. The mean NAD(P)H a2% fraction—reflecting the protein-bound NADH component—was 22.31±0.32% in the WT group and 25.58±0.71% in the PS19 group (mean ± SD calculated from n=5 WT and n=4 PS19 animal-level means, to account for the nested structure of pixel-level measurements within each animal). Despite the modest absolute difference (∼2.50 percentage points), the extraordinarily large sample sizes yielded sufficient statistical power to detect this difference with high confidence. The Welch’s t-statistic was −8.51 and the two-tailed p value was 0.0011.

These results indicate a statistically significant elevation of the bound NAD(P)H fraction in PS19 neurons relative to wild-type controls, suggesting a shift toward increased enzyme-bound NADH and, by extension, altered mitochondrial metabolic activity. In the context of tauopathy, this finding is consistent with reported metabolic dysfunction in PS19 mouse models, where compromised oxidative phosphorylation efficiency may lead to a redistribution of the free-to-bound NADH ratio as detected by FLIM.^28^

Statistical analysis indicated an increase in $\mathrm{Ru}(\mathrm{BPY}{)}_{3}$ lifetime ($\tau_{1}$) in capillaries and veins of PS19 mouse cortex as compared with WT, with a smaller, nonsignificant increase observed in arteries. Segmentation of vascular networks were performed to identify the variation in the oxygen level in the capillaries (g), arteries (h), and veins (i). To further characterize differences in vascular oxygenation across cortical compartments, Welch’s two-sample t tests (assuming unequal variances) were performed separately for arteries, veins, and capillaries, though statistical comparisons were performed on animal-level mean values (n=5 WT, n=4 PS19) rather than pooled pixels, to avoid pseudoreplication. Vascular structures were manually selected across multiple fields of view (FOVs) acquired from the cortical surface, with the number of structures per FOV varying depending on the vascular density and imaging area sampled. In total, 6 arteries, 20 veins, and 30 capillaries were identified and segmented in WT mice, compared with 16 arteries, 40 veins, and 20 capillaries in PS19 mice, refined from the initial segmentation to exclude vessels with ambiguous wall boundaries or insufficient photon counts for reliable lifetime fitting; capillary segments were manually delineated as single vessels up to the point of branching, based on visual assessment. The variation in the number of selected vessels across animals and groups reflects the heterogeneity of cortical vascular architecture sampled within each FOV rather than a systematic bias in vessel selection. In arteries, $\tau_{1}$ was 320.10±13.33  ns in WT and 335.98±20.51  ns in PS19 (t=−1.34, p=0.239). In veins, $\tau_{1}$ was 485.59±17.51  ns in WT and 579.85±22.69  ns in PS19 (t=−6.84, p=0.0006). In capillaries, $\tau_{1}$ was 420.48±10.71  ns in WT and 452.94±17.97  ns in PS19 (t=−3.19, p=0.027). In capillaries and veins, PS19 mice exhibited significantly prolonged ruthenium $\tau_{1}$ values relative to WT controls (p=0.027 and p=0.0006, respectively); the arterial difference did not reach statistical significance (p=0.239) at this sample size, although the direction of change was consistent with reduced oxygen quenching of the ruthenium probe and indicative of decreased intravascular oxygen levels throughout the cortical vasculature.

The increased lifetime percentage correlates with lower oxygen levels in the vascular areas of PS19 mouse cortex than WT. This suggests that PS19 vasculature has an oxygen deficit versus Intravascular oxygen tension measurements in WT mice revealed that capillary ${\mathrm{pO}}_{2}$ values surpassed those observed in arteries and veins. By contrast, PS19 transgenic mice demonstrated systemic microvascular hypoxia, characterized by pronounced reductions in ${\mathrm{pO}}_{2}$ throughout the vascular hierarchy compared with age-matched WT controls. Compared with WT controls, PS19 mice showed a progressive reduction in cortical ${\mathrm{pO}}_{2}$ across all vessel types. Arterial ${\mathrm{pO}}_{2}$ decreased from 81.46±6.65 to 74.28±9.22  mmHg (t=1.31, p=0.244). Capillary ${\mathrm{pO}}_{2}$ showed a larger decline from 34.99±3.51 to 25.45±5.02 mmHg (t=3.23, p=0.022), reflecting impaired oxygen delivery at the microvascular level. The most pronounced deficit was observed in venous ${\mathrm{pO}}_{2}$, which fell from 18.84±2.44 to 8.32±2.16  mmHg (t=6.86, p=0.0003), indicating substantially increased oxygen extraction or impaired venous oxygenation in PS19 mice. The progressive amplification of the ${\mathrm{pO}}_{2}$ deficit from artery to capillary to vein is consistent with cumulative microvascular dysfunction and elevated tissue oxygen demand in the PS19 tauopathy model, with significantly reduced ${\mathrm{pO}}_{2}$ in capillaries and veins relative to WT controls; the arterial difference did not reach statistical significance at this sample size (p=0.244).

## 6 Discussion and Conclusions

In this paper, we have applied 2P- FLIM and - PLIM to compare metabolic states of anesthetized live WT and PS19 mice, based on open, cranial window imaging. We took advantage of auto-fluorescent properties of coenzymes NAD(P)H tracking metabolic states and oxygen tension metrics recorded by the $\mathrm{Ru}(\mathrm{BPY}{)}_{3}$ oxygen sensing probe. The enzyme bound NAD(P)H a2[%] is a key metabolic indicator of mitochondrial activity and early metabolic disruptions in neurodegenerative diseases.^8^^,^^9^^,^^11^ As energy metabolism was the primary focus of this study, NAD(P)H a2% was compared between WT and PS19 mice under anesthetized resting-state conditions, revealing a significantly higher level of mitochondrial metabolic activity in PS19 relative to WT. Increased enzyme-bound NAD(P)H fraction has been similarly reported in primary neurons from transgenic AD mouse models, reflecting enhanced mitochondrial oxidative activity.^43^^,^^44^

The ruthenium probe has been successfully applied as an oxygen sensing probe in previous biomedical studies.^26^^,^^39^^,^^45^ Probe concentration was optimized prior to *in vivo* imaging as described in the Methods section. PLIM microscopy provided valuable insight into cortical ${\mathrm{pO}}_{2}$ differences between WT and PS19 mice. The ruthenium probe demonstrated linear Stern-Volmer oxygen dependence across microenvironments, enabling quantitative ${\mathrm{pO}}_{2}$ measurements. For live imaging applications, lifetime of $\mathrm{Ru}(\mathrm{BPY}{)}_{3}$ not only influenced by the presence of oxygen but also a function of binding with biomolecules, extravascular region after leakage, electrostatic interactions with the inner wall of blood vessels.^37^^,^^46^^,^^47^ Quantitative analysis reveals the bi-exponential nature of $\mathrm{Ru}(\mathrm{BPY}{)}_{3}$ lifetime in mouse cortex where $\tau_{1}$ component is sensitive to oxygen concentration as compared with $\tau_{2}$. $\mathrm{Ru}(\mathrm{BPY}{)}_{3}$ lifetime ($\tau_{1}$) was significantly increased in PS19 capillaries and veins relative to WT, with a smaller, nonsignificant increase in arteries. Veins showed a ∼19% increase in $\tau_{1}$ in PS19 relative to WT (p=0.0006), whereas the arterial increase (∼5%) did not reach statistical significance (p=0.239). Capillaries showed a ∼7.7% increase in $\tau_{1}$ in PS19 relative to WT (p=0.027). PS19 mice exhibited progressive vascular hypoxia across vessel types, with ${\mathrm{pO}}_{2}$ reductions of ∼8.8% in arteries, ∼27.3% in capillaries, and ∼55.8% in veins compared with WT controls, consistent with cumulative oxygen depletion along the vascular tree; the arterial reduction did not reach statistical significance at this sample size (p=0.244), whereas the capillary and venous reductions remained statistically robust (p=0.022 and p=0.0003, respectively). The venous ${\mathrm{pO}}_{2}$ reduction should be interpreted with caution as the PS19 venous $\tau_{1}$ approaches $\tau_{0}$, reducing probe sensitivity at this oxygen range.

The present study has certain limitations regarding its scope, as measurements were performed under anesthesia to ensure stable cranial window imaging conditions. A limitation of the current ${\mathrm{pO}}_{2}$ quantification approach is that Stern-Volmer calibration constants ($k_{q}$ and $\tau_{0}$) were derived from *in vitro* measurements using $\mathrm{Ru}(\mathrm{bpy}{)}_{3}^{2+}$ in aqueous solution and directly applied to *in vivo* $\tau_{1}$ values. This assumes that the photophysical properties of the probe are not substantially altered by the intravascular environment, including differences in viscosity, protein binding, and local pH. Although calibration was performed at physiological temperature (37°C) to minimize thermal effects, future studies should consider *in situ* validation using an independent ${\mathrm{pO}}_{2}$ measurement such as a Clark electrode to confirm calibration accuracy *in vivo*.^48^ The ruthenium probe concentration was optimized at 0.5 mM following careful titration to balance signal quality and biocompatibility. The current study represents a cross-sectional comparison between WT and PS19 mice, providing a foundation for future longitudinal studies tracking metabolic and vascular changes across disease progression. The sample size (n=5 WT and n=4 PS19) is consistent with established *in vivo* cranial window imaging studies, where surgical complexity limits cohort size. Future studies incorporating larger cohorts would further consolidate these findings.

Thus, we conclude that simultaneous FLIM-PLIM imaging enables comprehensive characterization of cerebral metabolism and vascular oxygenation in PS19 mice, which can be applied to a more exhaustive protocol, beyond the limits of this report.

## Supplementary Material

10.1117/1.NPh.13.3.035008.s01

## Data Availability

The authors have declared that the availability of data used in the research results reported in the manuscript will be provided by contacting the corresponding author.

## References

[1] Raichle M. E. Gusnard D. A., “Appraising the brain’s energy budget,” Proceed. Natl. Acad. Sci. U. S. A. 99(16), 10237–10239 (2002). 10.1073/pnas.172399499 PMC124895 12149485

[2] Watts M. E. Pocock R. Claudianos C., “Brain energy and oxygen metabolism: emerging role in normal function and disease,” Front. Mol. Neurosci. 11, 216 (2018). 10.3389/fnmol.2018.00216 29988368 PMC6023993

[3] Ahmad M. et al., “Electron transport chain,” in StatPearls, StatPearls Publishing LLC (2025). 30252361

[4] Epstein T. et al., “Separation of metabolic supply and demand: Aerobic glycolysis as a normal physiological response to fluctuating energetic demands in the membrane," Cancer Metab. 2(1), 7 (2014). 10.1186/2049-3002-2-7 24982758 PMC4060846

[5] Zhang X. Alshakhshir N. Zhao L., “Glycolytic metabolism, brain resilience, and Alzheimer’s disease,” Front. Neurosci. 15, 662242 (2021). 10.3389/fnins.2021.662242 33994936 PMC8113697

[6] Schaefer P. M. et al., “NADH autofluorescence—a marker on its way to boost bioenergetic research,” Cytometry Part A 95(1), 34–46 (2019). 10.1002/cyto.a.23597 30211978

[7] Periasamy A. Clegg R. M., FLIM Microscopy in Biology and Medicine, CRC Press, Boca Raton, Florida (2009).

[r8] Sagar V. K. et al., In Vivo Two-Photon FLIM Imaging to Investigate Metabolism in Live Animals, Vol. 68. SPIE, 2024).

[r9] Norambuena Aés Wallrabe H. Cao R. Wang D. B. Silva A. Svindrych Z. Periasamy A. Hu S. Tanzi R. E. Kim D. Y. Bloom G. S., “A novel lysosome-to-mitochondria signaling pathway disrupted by amyloid-β oligomers,” EMBO J. 37(22), e100241 (2018). 10.15252/embj.2018100241 30348864 PMC6236329

[10] Gómez C. A., “Cerebral metabolism in a mouse model of Alzheimer’s disease characterized by two-photon fluorescence lifetime microscopy of intrinsic NADH,” Neurophotonics 5(4), 045008 (2018). 10.1117/1.NPh.5.4.045008 30603656 PMC6307680

[11] Norambuena A. et al., “Disrupted mitochondrial response to nutrients is a presymptomatic event in the cortex of the APPSAA knock-in mouse model of Alzheimer’s disease,” Alzheimer’s Dement. 20(10), 6844–6859 (2024). 10.1002/alz.14144 39171353 PMC11485302

[12] Niederschweiberer M. A. et al., “NADH fluorescence lifetime imaging microscopy reveals selective mitochondrial dysfunction in neurons overexpressing alzheimer’s disease–related proteins,” Front. Mol. Biosci. 8, 671274 (2021). 10.3389/fmolb.2021.671274 34195227 PMC8236706

[13] Sakadžić S. et al., “Two-photon high-resolution measurement of partial pressure of oxygen in cerebral vasculature and tissue,” Nat. Methods 7(9), 755–759 (2010). 10.1038/nmeth.1490 20693997 PMC2932799

[14] Wheaton W. W. Chandel N. S., “Hypoxia. 2. Hypoxia regulates cellular metabolism,” Am. J. Physiol. Cell Physiol. 300(3), C385–C393 (2011). 10.1152/ajpcell.00485.2010 21123733 PMC3063979

[15] Mosconi L. et al., “Multicenter standardized 18F-FDG PET diagnosis of mild cognitive impairment, Alzheimer’s disease, and other dementias,” J. Nucl. Med. 49(3), 390–398 (2008). JNMEAQ 0161-5505 10.2967/jnumed.107.045385 18287270 PMC3703818

[16] Frackowiak R. S. Friston K. J., “Functional neuroanatomy of the human brain: positron emission tomography—a new neuroanatomical technique,” J. Anat. 184(Pt 2), 211–225 (1994). JOANAY 0021-8782 8014115 PMC1259983

[17] Ferrari M. Quaresima V., “A brief review on the history of human functional near-infrared spectroscopy (fNIRS) development and fields of application,” NeuroImage 63(2), 921–935 (2012). NEIMEF 1053-8119 10.1016/j.neuroimage.2012.03.049 22510258

[18] Khan N. et al., “Repetitive tissue ${\mathrm{pO}}_{2}$ measurements by electron paramagnetic resonance oximetry: current status and future potential for experimental and clinical studies,” Antioxid. Redox Signal. 9(8), 1169–1182 (2007). 10.1089/ars.2007.1635 17536960 PMC2921178

[19] Logothetis N. K. Pauls J. Augath M. Trinath T. Oeltermann A., “Neurophysiological investigation of the basis of the fMRI signal,” Nature 412(6843), 150–157 (2001). 10.1038/35084005 11449264

[20] Parpaleix A. Houssen Y. G. Charpak S., “Imaging local neuronal activity by monitoring ${\mathrm{pO}}_{2}$ transients in capillaries,” Nat. Med. 19(2), 241–246 (2013).1078-8956 10.1038/nm.3059 23314058

[r21] Becker W. et al., “Simultaneous fluorescence and phosphorescence lifetime imaging,” Proc. SPIE 7903, 790320 (2011). 10.1117/12.875204

[r22] Esipova T. V. et al., “Oxyphor 2P: a high-performance probe for deep-tissue longitudinal oxygen imaging,” Cell Metab. 29(3), 736–744 (2019). 10.1016/j.cmet.2018.12.022 30686745 PMC6402963

[r23] Erlebach E. et al., “Measurement of cerebral oxygen pressure in living mice by two-photon phosphorescence lifetime microscopy,” STAR Protoc. 3(2), 101370 (2022). 10.1016/j.xpro.2022.101370 35573482 PMC9092998

[r24] Kalinina S. et al., “Correlative NAD(P)H-FLIM and oxygen sensing-PLIM for metabolic mapping,” J. Biophoton. 9(8), 800–811 (2016). 10.1002/jbio.201500297 26990032

[r25] Parshina Y. P. et al., “Simultaneous probing of metabolism and oxygenation of tumors in vivo using FLIM of NAD(P)H and PLIM of a new polymeric Ir(III) oxygen sensor,” Int. J. Mol. Sci. 23(18), 10263 (2022).1422-0067 10.3390/ijms231810263 36142177 PMC9499414

[26] Shcheslavskiy V. I. et al., “Combined fluorescence and phosphorescence lifetime imaging, Appl. Phys. Lett. 108(9) (2016). 10.1063/1.4943265

[27] www.jax.org

[28] Pardo E. Kim T. Wallrabe H. Zengeler K. E. Sagar V. K. Mingledorff G. Sun X. Periasamy A. Lukens J. R. Bloom G. S. Norambuena Aés, “Mitochondrial NADK2-dependent NADPH controls tau oligomer uptake in human neurons,” Alzheimer’s Dement. 22(6) (2026). 10.1002/alz.71594 PMC13272107 42304136

[29] www.lsmtech.com

[30] www.becker-hickl.com

[31] Becker W., The bh TCSPC Handbook, 11th ed., Becker & Hickl GmbH, Berlin (2026).

[32] Hosny N. A. Lee D. A. Knight M. M., “Single photon counting fluorescence lifetime detection of pericellular oxygen concentrations,” J. Biomed. Opt. 17(1), 016007 (2012). 10.1117/1.JBO.17.1.016007 22352657

[33] Dmitriev R. I., Multi-Parametric Live Cell Microscopy of 3D Tissue Models, Springer (2017). 10.1007/978-3-319-67358-5_4 29080130

[34] Morris K. J. et al., “Luminescence lifetime standards for the nanosecond to microsecond range and oxygen quenching of ruthenium(II) complexes,” Anal. Chem. 79(24), 9310–9314 (2007). ANCHAM 0003-2700 10.1021/ac0712796 17985845

[35] www.biospherix.com

[36] Tsuneyasu S. et al., “Ultrafast response in AC-driven electrochemiluminescent cell using electrochemically active $\mathrm{DNA}/\mathrm{Ru}(\mathrm{bpy}{)}_{3}^{2+}$ hybrid film with mesoscopic structures,” Sci. Rep. 7(1), 8525 (2017). 10.1038/s41598-017-09123-2 28819318 PMC5561079

[37] Pyle A. M. et al., “Mixed-ligand complexes of ruthenium(II): factors governing binding to DNA,” J. Am. Chem. Soc. 111(8), 3051–3058 (1989). JACSAT 0002-7863 10.1021/ja00190a046

[38] Sun J. et al., “Ruthenium (II) complexes interact with human serum albumin and induce apoptosis of tumor cells,” Biol. Trace Elem. Res. 163(1-2), 266–274 (2015). 10.1007/s12011-014-0165-7 25398541

[39] Huntosova V. Gay S. Nowak-Sliwinska P. Rajendran S. K. Zellweger M. van den Bergh H. Wagnières G., “In vivo measurement of tissue oxygenation by time-resolved luminescence spectroscopy: advantageous properties of dichlorotris(1, 10-phenanthroline)-ruthenium(II) hydrate,” J. Biomed. Opt. 19(7), 077004 (2014). 10.1117/1.jbo.19.7.077004 25036215

[40] Zhou K. Du L. Ding R. Xu L. Shi S. Wang S. Wang Z. Zhang G. He G. Zhao Z. Tang B. Z., “Photocatalytic therapy via photoinduced redox imbalance in biological system,” Nat. Commun. 15(1), 10551 (2024). 10.1038/s41467-024-55060-w 39632877 PMC11618361

[41] Li H. et al., “In vivo identification of arteries and veins using two-photon excitation elastin autofluorescence, J. Anat. 236(1), 171–179 (2020). 10.1111/joa.13080 31468540 PMC6904638

[42] TeSlaa T. Ralser M. Fan J. Rabinowitz J. D., “The pentose phosphate pathway in health and disease,” Nat. Metab. 5(8), 1275–1289 (2023). 10.1038/s42255-023-00863-2 37612403 PMC11251397

[43] Clark L. C. et al., “Continuous recording of blood oxygen tensions by polarography, J. Appl. Physiol. 6(3), 189–193 (1953). 10.1152/jappl.1953.6.3.189 13096460

[44] Dong Y. et al., “Reversibility of age-related oxidized free NADH redox states in alzheimer’s disease neurons by imposed external Cys/CySS redox shifts,” Sci. Rep. 9(1), 11274 (2019). 10.1038/s41598-019-47582-x 31375701 PMC6677822

[45] Babilas P. et al., “In vivo phosphorescence imaging of ${\mathrm{pO}}_{2}$ using planar oxygen sensors,” Microcirculation 12(6), 477–487 (2005). MCCRD8 0275-4177 10.1080/10739680591003314 16147465

[46] Wang W. J. et al., “Induction and monitoring of DNA phase separation in living cells by a light-switching ruthenium complex,” J. Am. Chem. Soc. 143(30), 11370–11381 (2021). 10.1021/jacs.1c01424 34291952

[47] Wu C. H. et al., “Functionalizing collagen with vessel-penetrating two-photon phosphorescence probes: a new in vivo strategy to map oxygen concentration in tumor microenvironment and tissue ischemia,” Adv. Sci. 8(20), e2102788 (2021). 10.1002/advs.202102788 PMC8529487 34414696

[48] Clark L. C. et al., “Continuous recording of blood oxygen tensions by polarography,” J. Appl. Phys. 6(3), 189 (1953). 10.1152/jappl.1953.6.3.189 13096460

