# Supplementary material for: Metabolism and oxygen tension in Alzheimer’s disease mouse model using two-photon FLIM-PLIM microscopy
Source: Neurophotonics. 2026 Sep 11;13(3):035008. doi: 10.1117/1.NPh.13.3.035008 (PMC13566223; doi:10.1117/1.NPh.13.3.035008)
Supplement: Supplementary file 1 [file NPh_013_035008_SD001.pdf]

Supplementary Information

Metabolism and oxygen tension in Alzheimer's disease mouse model using 2-photon FLIM-PLIM microscopy

Vijay Kumar Sagar[a], Horst Wallrabe[a], Evelyn Pardo[b], Shagufta Rehman Alam[a], Wolfgang Becker[d], Andrés Norambuena[b]*, Ammasi Periasamy[a,b,c]*

University of Virginia, The W.M. Keck Center for Cellular Imaging[a], Department of Biology[b], Department of Biomedical Engineering[c], Charlottesville, USA

Becker & Hickl, GmbH[d], Nahmitzer Damm 30, 12277 Berlin, Germany

Supplementary

S1: Power-dependent assessment of cortical tissue integrity during two-photon imaging.

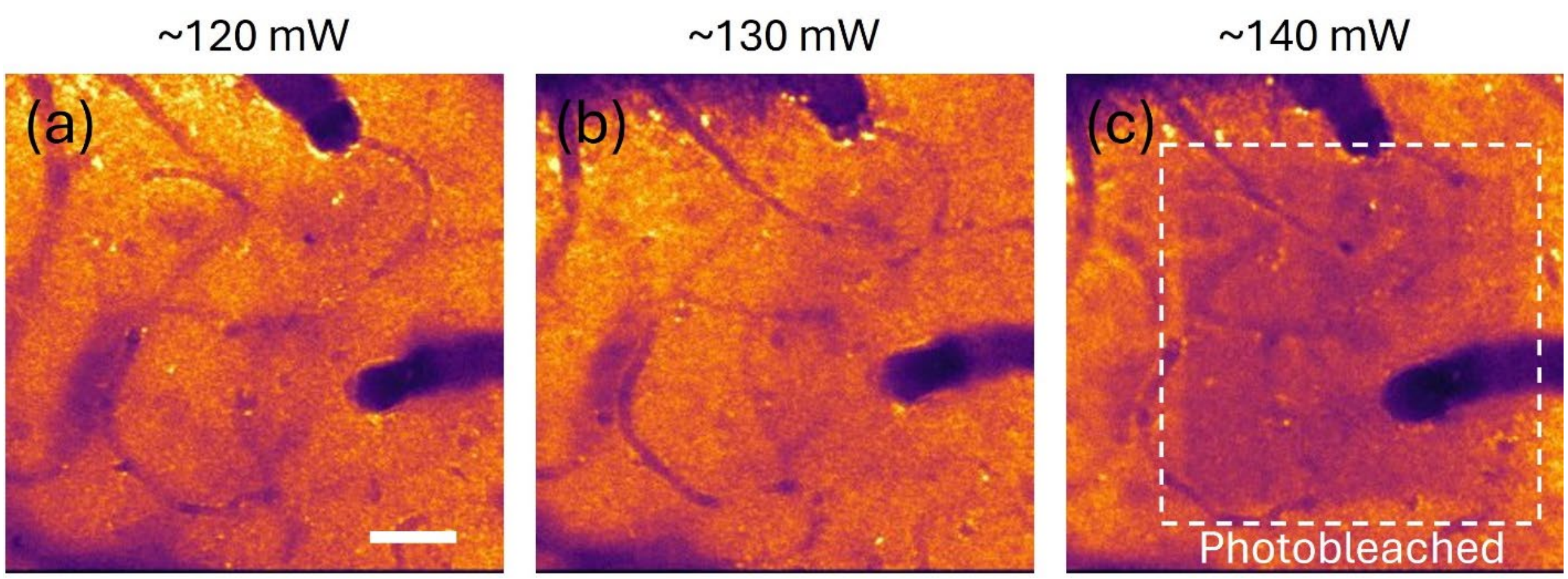

Figure S1: In vivo two-photon intensity images of mouse cortex acquired at excitation powers of ~120 mW (a), ~130 mW (b), and ~140 mW (c) at the objective output, using a Zeiss Plan-Apochromat 10×/0.45 objective at 800 nm excitation wavelength. Photobleaching is visible at ~140 mW as a distinct bleached region highlighted by the dashed white box in panel (c). All experimental imaging was performed using laser power below the photobleaching threshold. Any sub-threshold photobleaching not immediately visible to the eye would be comparable factor present equally in both WT and PS19 control images and is therefore unlikely to introduce systematic bias in the reported measurements. Scale bar 50 μm.

S2: Robustness of NAD(P)H fluorescence lifetime fitting across photon count regimes

To demonstrate the robustness of bi-exponential tail fitting across photon count regimes, representative NAD(P)H fluorescence decay histograms from three ROIs in mouse cortex with differing photon counts are shown in Supplementary Figure S2.

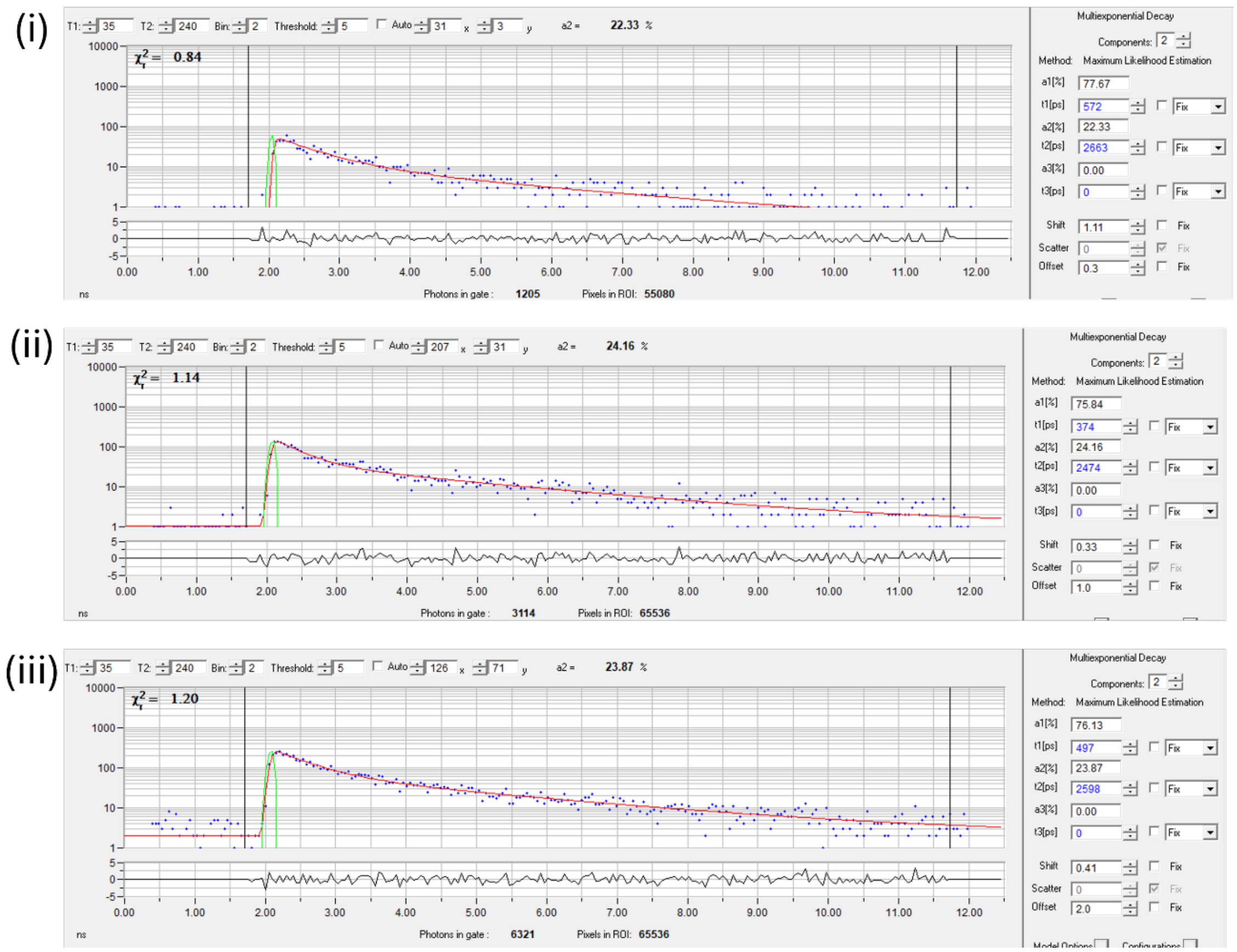

Figure S2. NAD(P)H fluorescence lifetime decay fitting across photon count regimes in mouse cortex. Representative bi-exponential decay histograms (blue dots) with bi-exponential fits (red curves) and instrument response function (green curve) from three ROIs containing 1,205 (a), 3,114 (b), and 6,321 (c) photons in gate respectively. Weighted residuals are shown below each decay. Fitting was performed using maximum likelihood estimation (SPCImage, Becker & Hickl). χ^2_r values are indicated on each panel.

The enzyme-bound fraction a2% remained stable within a narrow range (22.33-24.16%) across a five-fold difference in photon counts (1,205-6,321 photons/gate), and the long lifetime component τ_2 varied minimally (2,474-2,663 ps), confirming fitting robustness. Minor variation in τ_1 (374-572 ps) is expected as the short free NAD(P)H lifetime component is more sensitive to photon statistics at lower counts. Regional heterogeneity in NAD(P)H binding reflects genuine metabolic diversity across cortical cell populations rather than a fitting artefact.

S3: Two-component ruthenium Ru(BPY)₃ lifetime fitting

The 2-component PLIM fitting yields τ_1 (400-600 ns) and τ_2 (~2000 ns) across all vascular compartments. The τ_2 values (~2000 ns) are exceptionally prolonged compared to reported in vitro and in vivo ruthenium lifetime values, suggesting probe association with biomolecules rather than free intravascular oxygen sensing. Therefore, τ_1 represents the physiologically relevant oxygen-quenched component and was used exclusively for pO2 quantification via the Stern-Volmer relationship.

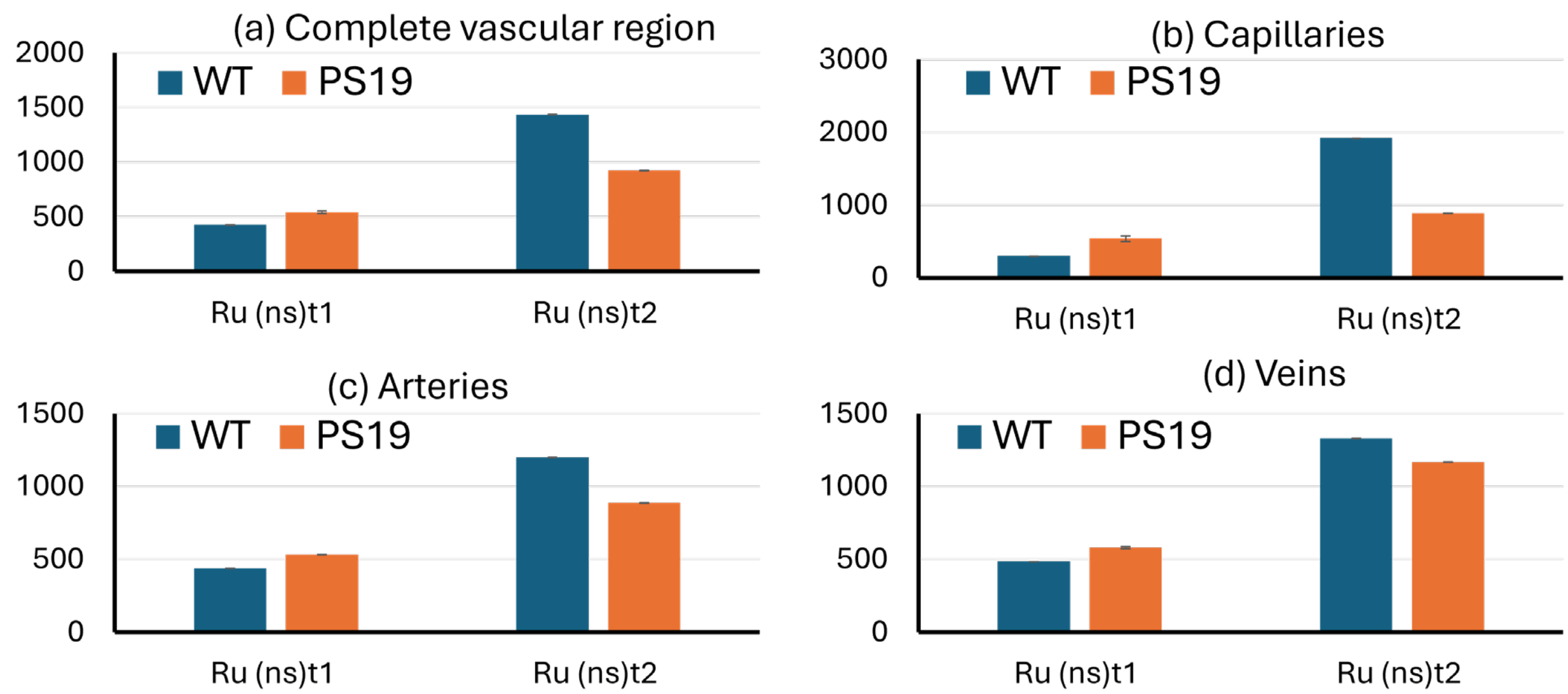

Figure S3. Two-component ruthenium Ru(BPY)₃ lifetime fitting parameters τ_1 and τ_2 for WT and PS19 mice across cortical vascular compartments: (a) complete vascular region, (b) capillaries, (c) arteries, and (d) veins.
